# Irreversible Electroporation for the Ablation of Renal Cell Carcinoma: A Prospective, Human, In Vivo Study Protocol (IDEAL Phase 2b)

**DOI:** 10.2196/resprot.6725

**Published:** 2017-02-16

**Authors:** Mara Buijs, Krijn P van Lienden, Peter GK Wagstaff, Matthijs JV Scheltema, Daniel M de Bruin, Patricia J Zondervan, Otto M van Delden, Ton G van Leeuwen, Jean JMCH de la Rosette, M Pilar Laguna

**Affiliations:** ^1^ Academic Medical Center Department of Urology University of Amsterdam Amsterdam Netherlands; ^2^ Academic Medical Center Department of Radiology University of Amsterdam Amsterdam Netherlands; ^3^ Academic Medical Center Department of Biomedical Engineering and Physics University of Amsterdam Amsterdam Netherlands

**Keywords:** irreversible electroporation, IRE, ablation, kidney, renal cell carcinoma, cancer, safety, efficacy

## Abstract

**Background:**

Irreversible electroporation (IRE) is an emerging technique delivering electrical pulses to ablate tissue, with the theoretical advantage to overcome the main shortcomings of conventional thermal ablation. Recent short-term research showed that IRE for the ablation of renal masses is a safe and feasible treatment option. In an ablate and resect design, histopathological analysis 4 weeks after radical nephrectomy demonstrated that IRE-targeted renal tumors were completely covered by ablation zone. In order to develop a validated long-term IRE follow-up study, it is essential to obtain clinical confirmation of the efficacy of this novel technology. Additionally, follow-up after IRE ablation obliges verification of a suitable imaging modality.

**Objective:**

The objectives of this study are the clinical efficacy and safety of IRE ablation of renal masses and to evaluate the use of cross-sectional imaging modalities in the follow-up after IRE in renal tumors. This study conforms to the recommendations of the IDEAL Collaboration and can be categorized as a phase 2B exploration trial.

**Methods:**

In this prospective clinical trial, IRE will be performed in 20 patients aged 18 years and older presenting with a solid enhancing small renal mass (SRM) (≤4 cm) who are candidates for ablation. Magnetic resonance imaging (MRI) and contrast-enhanced ultrasound (CEUS) will be performed at 1 day pre-IRE, and 1 week post-IRE. Computed tomography (CT), CEUS, and MRI will be performed at 3 months, 6 months, and 12 months post-IRE.

**Results:**

Presently, recruitment of patients has started and the first inclusions are completed. Preliminary results and outcomes are expected in 2018.

**Conclusions:**

To establish the position of IRE ablation for treating renal tumors, a structured stepwise assessment in clinical practice is required. This study will offer fundamental knowledge on the clinical efficacy of IRE ablation for SRMs, potentially positioning IRE as ablative modality for renal tumors and accrediting future research with long-term follow-up.

**Trial Registration:**

Clinicaltrials.gov registration number NCT02828709; https://clinicaltrials.gov/ct2/show/NCT02828709 (archived by WebCite at http://www.webcitation.org/6nmWK7Uu9). Dutch Central Committee on Research Involving Human Subjects NL56935.018.16

## Introduction

### Ablative Therapy in Renal Cell Carcinoma

Due to widespread detection of small renal masses (SRMs), a gradual but sustained rise in incidence of renal tumors 4 cm or less (cT1a, according to the TNM [tumor/node/metastasis] staging system) has been observed [1-4]. At present, the reference standard therapy in the management of SRMs is nephron sparing surgery like partial nephrectomy [5]. However, a significant interest is sparked in minimally invasive therapies, including cryoablation and radiofrequency ablation (RFA). Literature shows that thermal ablation compared to partial nephrectomy is characterized by a slightly higher recurrence rate but also accompanied by a lower complication rate [6,7]. Nevertheless, a growing body of research advocates that in selected patients similar oncological results can be obtained compared to those accomplished in surgical resection [8]. Current guidelines recommend primary ablative therapy in patients who are (1) not suitable for surgery, (2) have a genetic predisposition for developing multiple tumors, and (3) are diagnosed with bilateral tumors or have a solitary kidney and are at risk of complete loss of renal function after surgery [9-11].

Ablation of undesirable tissue depends on accurate dosing and adequate targeting of tumor destruction while sparing vital structures such as adjacent organs, collecting system, or major vessels [12,13]. Due to temperature fluctuations that are accompanied with the thermal character of cryoablation and RFA, it is thought that the destruction process of the tumor is unselective [14,15]. Ablation effects and tissue heating may be less effective in proximity to blood vessels as a result of thermal drainage by regional vascular flow impairing the extent of coagulation, in the literature termed as a “heat sink” effect [9,16]. Additionally, collateral damage to underlying vital structures can occur, as the natures of these structures are susceptible to extreme temperatures. Therefore according to guidelines, renal tumors located near the hilum or near the proximal ureter are not suitable for thermal ablation, forming a niche in ablative treatment of renal tumors [10].

### Irreversible Electroporation in Renal Cell Carcinoma

An emerging technique among the assortment of ablative modalities is called irreversible electroporation (IRE). It is based on high-voltage electrical pulses transferred between 2 or more needle electrodes. Charging the cell membrane causes holes in the membrane called nanopores, resulting in increased permeability of the cell and subsequent cell death [13,17-20].

Theoretically, the mechanism of action of IRE does not rely on temperature changes. Therefore it has been postulated that it has the potential to overcome the current limitations of thermal ablative modalities like cryoablation and RFA [12]. However, using the current clinical device settings, a rise in temperature is to be expected as shown by Wagstaff et al in an animal model [21].

With regard to IRE ablation in renal tumors, 4 studies have been performed in humans [20,22-24]. All studies concluded that safety of IRE in humans is warranted as long as electrocardiogram (ECG) synchronization is used.

Trimmer et al made a start in clinical efficacy, describing postablation features on cross-sectional imaging. Although these initial results seem promising and appear similar to conventional ablative techniques, a few limitations deserve consideration. The study design is retrospective, and the follow‐up was limited. Imaging was available in 15 out of 20 patients (75%) at 6-month follow-up and only in 6 out of 20 patients (30%) at 1-year follow-up [23].

Thomson et al performed IRE in various organs, including 10 renal tumors in 7 patients. One patient (14%) developed an ureteral stricture after IRE ablation in an area of the ureter that previously had been obstructed by RFA. Other centrally located tumors did not show any major complications. A total of 2 patients (29%) experienced minor complications consisting of transient hematuria [24]. Wendler et al were the first to provide histopathological data of IRE in renal tumors of 3 patients, showing complete coverage of the tumor within the ablation zone with preservation of the renal parenchyma [25]. Very small tumor residues of unclear malignant potential were found within the ablation zone. Unfortunately, clinical significance of these residues remained unclear and impossible to follow up since the tumors had been resected.

### Rationale

The first human studies have proven the safety of IRE for the ablation of SRMs. Initial results on clinical efficacy of IRE are promising and imply that effective oncological management is achievable. Clinical outcomes should be investigated in a small patient population to provide essential data before embarking on a randomized trial. We therefore plan to perform a study aiming at the clinical efficacy and safety of IRE in SRMs, with a specific focus on postablation follow-up with cross-sectional imaging. Research on IRE in liver tumors has demonstrated that ablation success of IRE decreases with tumor size above 4 cm [24]. According to current guidelines, ablative treatment is only offered to patients with SRMs (≤4 cm) [10]. Therefore, we aim to investigate IRE ablation in renal masses up to 4 cm. This prospective, human, in vivo trial is an essential step in order to safely progress to larger randomized trials on IRE of SRMs. This study conforms to the recommendations of the IDEAL (idea, development, exploration, assessment, long-term study) Collaboration and can be categorized as a phase 2B exploration trial [26] *.*

## Methods

### Study Objectives

- To determine the clinical efficacy of IRE ablation for SRMs (≤4 cm) assessed by the recurrence and residual disease rate at follow‐up using cross‐sectional imaging
- To evaluate the use of computed tomography (CT), magnetic resonance imaging (MRI), and contrast-enhanced ultrasound (CEUS) in the visualization of (non)complete ablation to assess the radiological extent of the ablation zone at 1 week, 3 months, 6 months, 9 months and 1 year after IRE
- To evaluate perioperative outcomes after IRE ablation of SRMs (≤4 cm) such as (1) renal function, measured by creatinine levels and estimated glomerular filtration rate (eGFR), (2) average length of hospital stay, (3) quality of life, and (4) postoperative pain score after IRE, measured by a visual analog scale (VAS) and analgesics use
- To determine the safety and feasibility of IRE ablation of SRMs (≤4 cm) by evaluating device and procedural adverse events using Common Terminology Criteria for Adverse Events (CTCAE) version 4.0

### Population

A total of 20 patients with solid enhancing SRMs on cross-sectional imaging qualifying for ablative therapy will be enrolled in this study. Eligible patients are 18 years of age and older and will receive a biopsy of the SRM before procedure. All inclusions are reviewed for safety and eligibility by a nephrologist participating in the research project. The inclusion and exclusion criteria for this study are listed in Textbox 1.

**Selection criteria. Severe cardiovascular disease is defined as the diagnosis of myocardial infarction, uncontrolled angina, significant ventricular arrhythmias, stroke or severe cardiac failure (New York Heart Association class III and IV) within 6 months prior to inclusion.**

Inclusion criteria:

- Age 18 years and older
- Solid enhancing SRM on cross-sectional imaging
- Candidate for ablation
- Signed informed consent

Exclusion criteria:

- Irreversible bleeding disorders
- Inability to stop anticoagulation therapy
- Implantable cardioverter-defibrillator or pacemaker
- Severe cardiovascular disease

### Study Design

**Figure 1:**
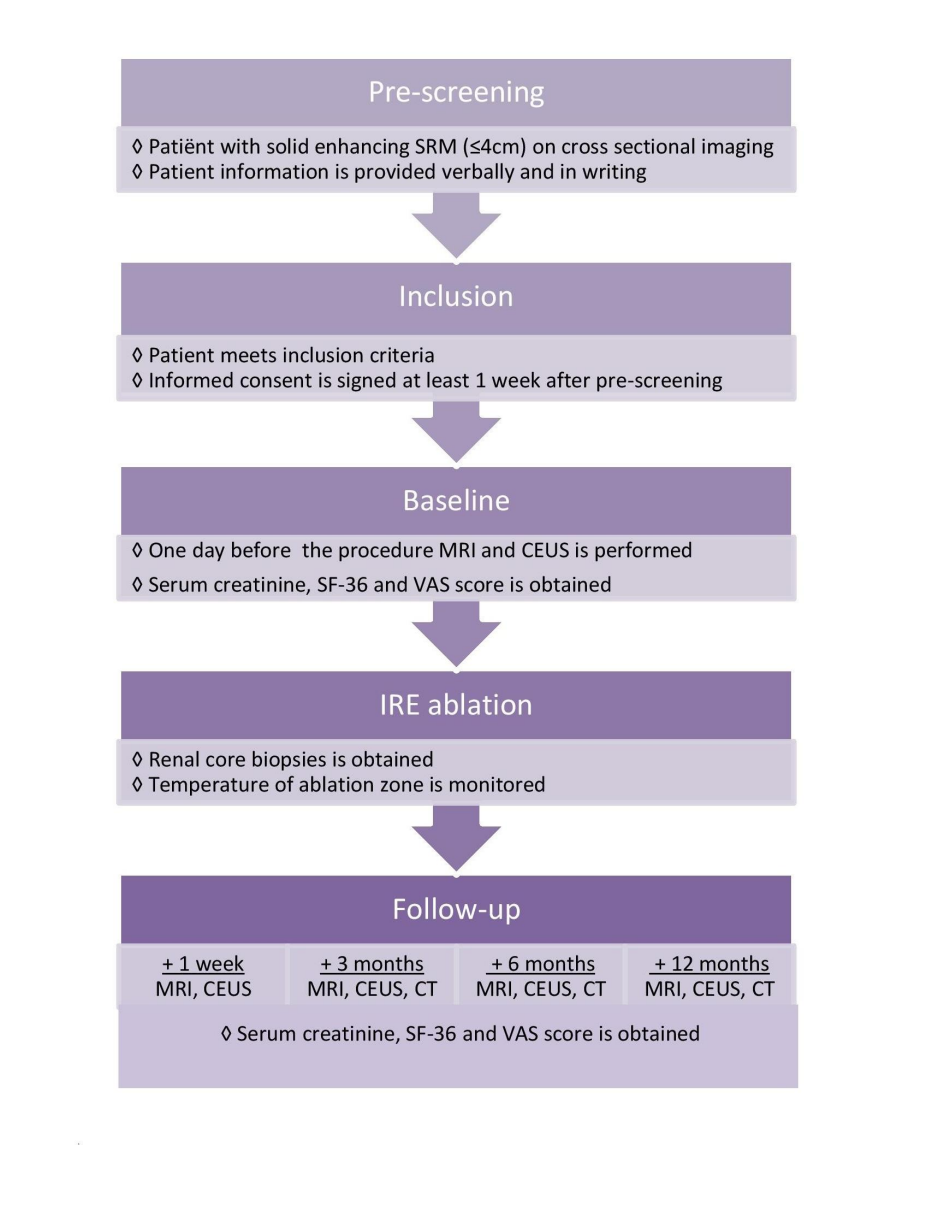
Study design flowchart.

This is a prospective, human, in vivo study among 20 patients presenting with solid enhancing SRM on cross-sectional imaging suspect for RCC. Preoperatively, imaging is required through CT, MRI, and CEUS. Furthermore, serum creatinine levels and VAS scores are obtained. A biopsy of the SRM will be performed prior to the ablation. IRE ablation will be performed using CT guidance, and ablation success will be measured directly after the ablation through contrast-enhanced CT. Device-related adverse events will be registered using the CTCAE version 4.0 guideline. At 1 week postablation, only CEUS and MRI will be performed to limit exposure to ionizing radiation. At 3 months, 6 months, and 12 months postablation, CEUS, MRI, and CT will be performed. Additionally at these time points, serum creatinine levels and VAS scores will be obtained, and quality of life will be assessed through Short Form 36 (SF-36) questionnaires. Residual and recurrent disease will be assessed through tissue enhancement on cross-sectional imaging. When imaging appears suspicious for recurrence or residual disease, a percutaneous renal core biopsy will be performed. A study flowchart demonstrating the investigations is outlined in Figure 1.

### Study Procedures

#### Renal Core Biopsy (Standard)

According to the ablation protocol of the Academic Medical Center University Hospital, percutaneous renal core biopsies will be obtained prior to the IRE procedure if patient desires. At least 2 core biopsies will be acquired for pathological examination.

#### Irreversible Electroporation Ablation (Study Intervention)

**Figure 2:**
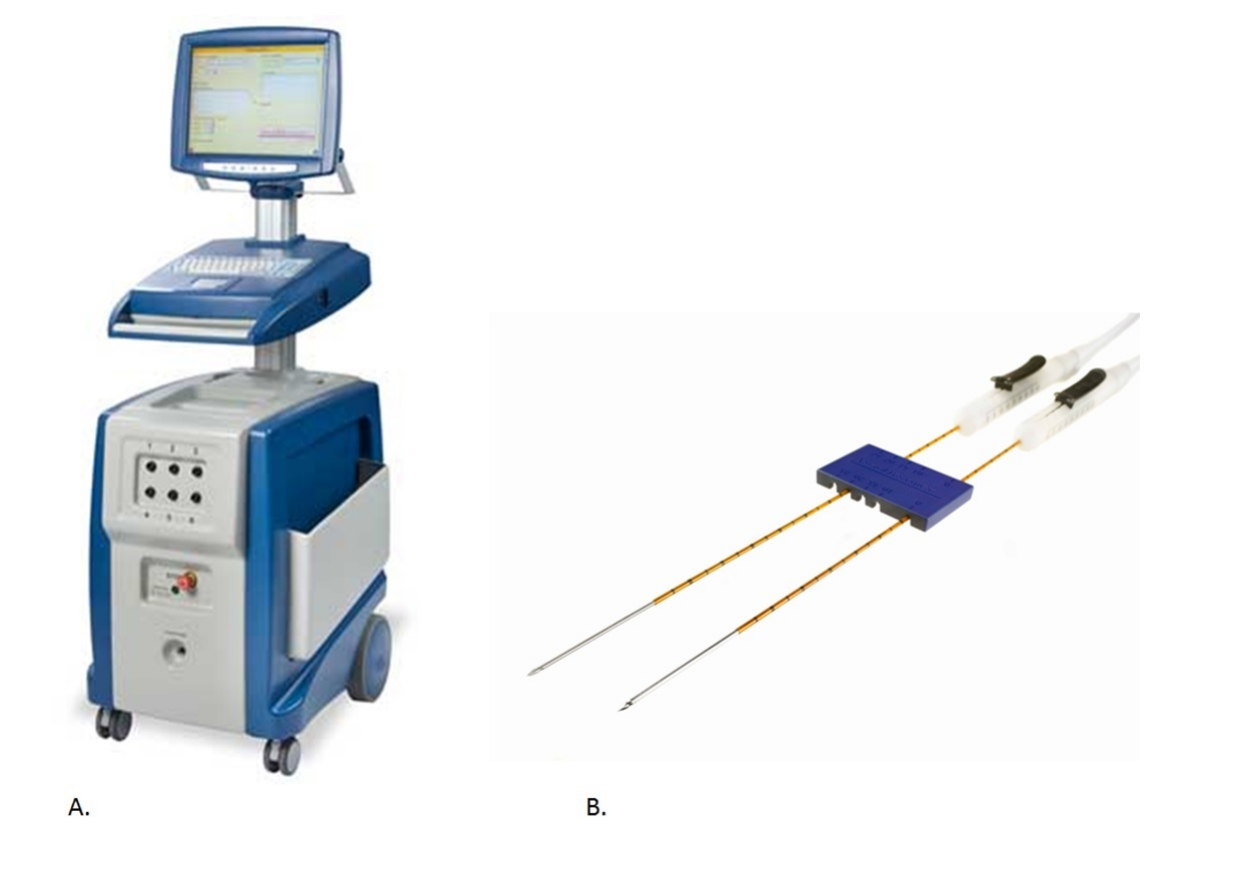
A. The NanoKnife IRE console. B. The console operates with 19G monopolar needle electrodes, which are bundled together using the external spacers.

In this study, IRE ablation is performed using the NanoKnife IRE device (AngioDynamics Inc) (Figure 2, A), also registered as the HVP-01 electroporation system. The IRE system contains a low energy direct current generator, a foot switch, and 19G monopolar needle electrodes (15 or 25 cm length). Regulatory authorities have approved both the device and the electrodes through a Conformité Européenne certificate for the use of cell membrane electroporation. Additionally, the US Food and Drug Administration has granted 510(k) clearance with premarket notifications (K060054, K080202, K080376, K080287). Granted 510(k) components are all approved for surgical ablation of soft tissue.

The IRE procedure will be performed at the radiology department CT room with the patient under general anesthesia including deep muscle relaxation to prevent severe muscle contraction [27]. CT imaging will be performed with the patient in the prone or lateral position, dependent on tumor location and position of adjacent organs such as intestines. An interventional radiologist cooperating with a urological surgeon, both experts on percutaneous ablative procedures, will perform the IRE procedure. IRE pulses will be synchronized with ECG under supervision of an anesthesiologist. Prior to ablation, a (second) set of biopsies will be obtained to confirm histopathology. Guided by CT and accompanied by an external spacer for fixation, needle electrodes will be placed (Figure 2, B). The amount of probes and probe placement will be attuned for specific tumor size and location, granting 15 mm between the electrodes with an active tip length of 15 mm. IRE pulses with pulse intensity of 1500 V/cm will be delivered in 90 consecutive pulses of 90 µs *.* Settings are used to disrupt the cell membrane potential in order to achieve irreversible permeability of the cell and subsequent apoptosis. Van den Bos et al demonstrated that with current settings the ablation zone is completely ablated without leaving any skip lesions within the electrode configuration [28].

The primary cycle of IRE will take 5 to 10 minutes with a total operating time (including anesthesia) of approximately 90 minutes. Immediately after IRE has been performed, a contrast-enhanced CT will be made to assess adequate ablation. It is expected that patients will be discharged 24 to 36 hours after the IRE procedure. Before patient’s discharge, quality of life and postprocedural pain will be assessed through SF-36 questionnaire and VAS score respectively. At 1 week after the procedure, VAS score and SF-36 questionnaire will be obtained and cross-sectional imaging by CEUS and MRI will be performed.

#### Computed Tomography Imaging (Standard)

As provided per ablation protocol, CT imaging will take place during the diagnostic phase and during the procedure. According to our ablation surveillance protocol, follow-up CT imaging will be performed at 3 months, 6 months, 1 year, 1.5 year, 2 years, 2.5 years, and 3 years after IRE ablation. After this, patients will be followed up yearly up to 10 years. This is the standardized follow‐up after ablative therapy at our institution (see Figure 1). Patients with an eGFR below 60 mL/min/1.73 m^2^ will undergo pre- and posthydration in order to prevent contrast-induced nephropathy. Patients with an eGFR below 30 mL/min/1.73 m^2^ are excluded from CT imaging.

CT imaging will be performed in a supine position in a dual source CT system, SOMATOM Force (Siemens Medical Solutions), or in a Sensation 64-slice CT scanner (Siemens Medical Solutions). First, a survey scan from the upper border of the diaphragm to the ischium bone will be made. Next, noncontrast series in the same section will be performed. Subsequently, 120 mL of Ultravist-300 diluted with NaCl 0.9% will be administered intravenously with a speed of 4 mL/s. Following contrast injection, arterial, venous, and delayed series will be obtained after 45 seconds, 115 seconds, and 600 seconds, respectively. Source images will be recontructed in coronal and sagittal planes using multiplanar reconstruction in the venous and delayed series.

#### Contrast-Enhanced Ultrasound and Magnetic Resonance Imaging (Study Intervention)

Baseline CEUS will take place 1 day before IRE, and 1 week, 3 months, 6 months, and 12 months after IRE. MRI imaging will take place 1 week, 3 months, 6 months, and 12 months post-IRE (see Figure 1). This frequency was established in order to assess lesion size and characteristics.

CEUS imaging encompasses microbubbles of 3 to 5 µm as a contrast agent to visualize blood flow. The phospholipid-coated microbubbles demonstrate regional tissue vascularization, including the tissue-specific microvasculature. This study uses a Philips iU22 (Phillips Healthcare) device united with a third-generation intravenous ultrasound contrast agent (SonoVue) for optimal imaging. Sonovue contrast agent is characterized by a distribution half-life of 1 minute and an elimination half-life of 6 minutes when intravenously administired [29].

MRI will be performed with the patient in the supine position using a 1.5 Tesla AVANTO MRI scanner (Siemens Healthcare) with a 16-channel body matrix array coil. According to our kidney tumor protocol, a minimum of 9 sequences will be performed: T2-trufi with fat suppression, T1-fl2d contrast-enhanced in and out of phase, T2-haste, T1-vibe unenhanced, and dynamic series at 30 seconds, 60 seconds, and 15 minutes **.** Intravenous contrast agent Gadovist (Bayer Pharma) of 0.1 mmol per kg of body weight will be administered for enhancement.

### Sample Size

Our sample size was deliberated on the basis of previous similar study designs that used comparable sample sizes of 6 to 20 patients [20,23-25]. In this phase of research (phase 2B IDEAL Collaboration), a small cohort of N=20 was chosen to acquire first results in order to progress to a large trial. A sample size of 20 patients does not permit reliable comparative statistical analysis. In this study, IRE is intended as a curative therapy. Consequently, there will be no exploration in number of probes and configuration settings. Hence, analysis will be restricted to averages and standard deviations of assessed radiologic features.

### Potential Benefits and Risks

Conventional focal ablative therapies, RFA and cryoablation, are indicated in patients presenting with an SRM who are poor surgical candidates or who are genetically predisposed to develop multiple tumors. For this study, IRE ablation will be offered to this group of patients in our institution. Early research into renal IRE has proven that the procedural safety and the periprocedural burden are comparable to conventional ablative therapies. The lack of long-term oncological follow-up poses a potential risk as patients cannot be counseled on the risk of residual or recurrent tumor. Post-IRE follow-up will be equal to postcryoablation and post-RFA follow-up and therefore does not carry additional burden with regard to ionizing radiation. When renal function appears to decrease to eGFR below 30 mL/min/1.73 m^2^, only MRI and CEUS will be performed to prevent contrast-induced nephropathy. Furthermore, potential risks associated with IRE ablation for renal tumors using the NanoKnife system are listed in Table 1.

**Table 2:** Potential risks associated with irreversible electroporation of renal tumors.

Potential hazards	Potential effects
Excessive energy delivery	Muscle contraction, burn, damage to critical anatomical structure, unintended tissue ablated, bradycardia/hypotension, vagal stimulation/asystole, electrical shock, myocardial infarction, stroke, death
Insufficient/no energy delivery	Ineffective ablation, no ablation
Unintended mains or patient circuit voltage exposure to patient or user	Electrical shock
Incorrect timing of pulse delivery	Transient arrhythmia, prolonged arrhythmia, stroke, death
Unintended interference with implanted devices containing electronics or metal parts	Myocardial infarction, stroke, death
Unexpected movement of the device and displacement of the electrodes	Hypotension, damage to critical anatomical structure, pneumothorax, mechanical perforation, hemorrhage, unintended tissue ablated, electrical shock, death
Sterile barrier breach	Infection, sepsis

### Data Safety Monitoring Board

The study will be monitored by a data safety monitoring board (DSMB) consisting of an independent urologist and a clinical epidemiologist. This team will monitor patient safety and treatment efficacy data during the study. Monitoring procedures are predetermined and described in the DSMB charter, approved by the institutional review board (IRB) of the Academic Medical Center University Hospital in Amsterdam. Additional DSMB meetings can be called at any time if deemed necessary by the DSMB or the principal investigator.

### Analysis

The NanoKnife console produces 2-dimensional images including a prediction of the ablation zone, which is perpendicular to the needle. The AMIRA (FEI) software package system will bundle the 2-dimensional ablation zone cross-sections around the length of the exposed tip. This will estimate the following:

- Ablation zone volume (cm^3^)
- 3-Dimensional reconstruction
- Ablation zone shape/symmetry

An experienced uroradiologist will evaluate CT and MRI images for the following characteristics *:*

- Volume of ablation zone (cm^3^)
- Shape of ablation zone
- Residual tumor on ablation zone border
- Skip lesions or signs of recurrence within ablation zone
- Transition zone between ablated and normal renal tissue
- Damage to adjacent vital structures

For MRI and CT, whole-mount kidney and ablation zone will be calculated. The AMIRA software system will be used to obtain a 3-dimensional kidney and ablation zone. CEUS will be performed by an interventional radiologist and will be used for 2-dimensional imaging only.

### Ethical Consideration

The IRB of the Academic Medical Center, Amsterdam, approved this study protocol (2016_055). The protocol has been registered with the Dutch Central Committee on Research Involving Human Subjects (NL56935.018.16) and is entered in the ClinicalTrials.gov database (NCT02828709). The study is conducted in accordance with the ethical principles and standards of Good Clinical Practice which have their origin in the Declaration of Helsinki (Fortaleza, Brazil, October 2013). Potential candidates will receive the study information both verbally and in writing. They will be granted at least 1 week to decide on participation. Written informed consent will be acquired from all participants. If deemed necessary, supplementary information will be provided verbally or in writing.

### Availability of Data and Materials

The study initiator, international coordinating researcher, and biostatistician have access to all data. All data is available for audit, and all data will be published in an international peer-reviewed medical journal. The datasets created in the current study are not publicly available due to protecting the privacy of participants but are available from the corresponding author on reasonable request.

## Results

**Figure 3:**
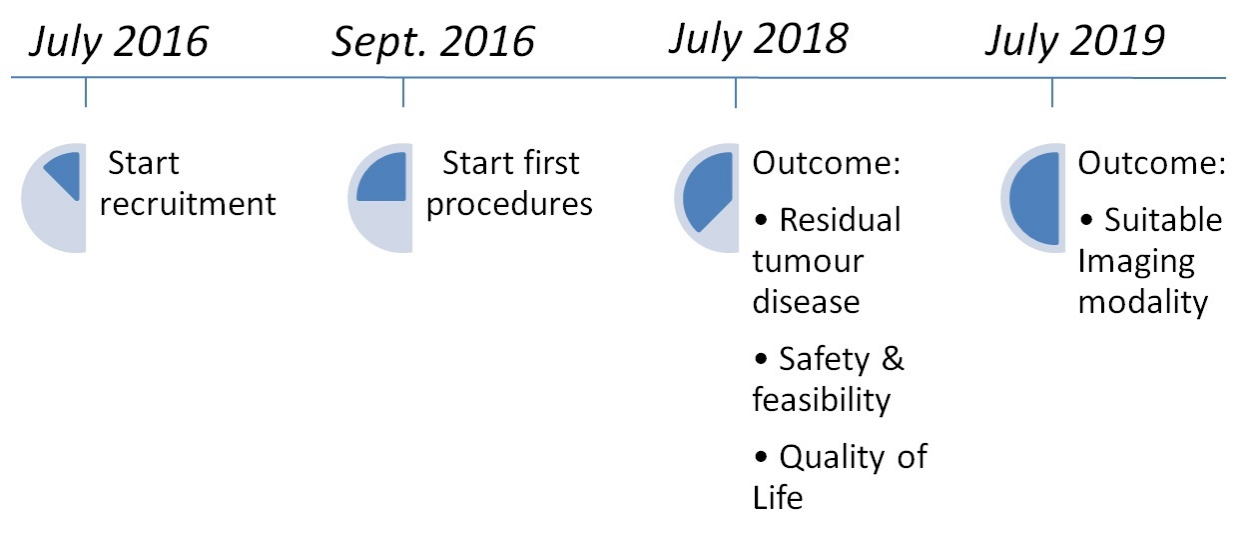
Planned timeline of recruitment, enrollment, and outcome.

At time of writing the trial is recruiting patients, with 2 inclusions completed. The expected inclusion rate is 1 patient every 6 weeks, resulting in an estimated inclusion period of 2 years. Hence we calculate that we will recruit the full sample size within 2 years. Additionally, early results on outcome of residual tumor disease, quality of life, and safety and feasibility will be acquired within 2 years (see Figure 3). The imaging follow-up in this study is 1 year for each patient (see Figure 1); therefore, we expect to complete the study in 2019.

## Discussion

### Principal Findings

IDEAL phase 1 and 2A research into IRE in renal tumors has shown encouraging short-term outcomes, paving the way for small-scale follow-up studies. In our opinion, it is crucial to investigate the clinical efficacy of IRE in renal tumors to serve as a solid base for a large randomized trial. We aim to determine the clinical effect of IRE by assessing the presence of enhancement on cross-sectional imaging during follow-up as it is advised in thermal ablation [30]. Whereas IRE is a novel ablation technology, posttreatment radiological features in CT scan or MRI are still ill-defined. However, retrospective preliminary research suggests the radiological pattern is similar to the one described after thermal ablation [23].

### Limitations

A limitation within our study is the absence of histopathological confirmation post-IRE. In literature, 2 in vivo studies have revealed the IRE ablation effects in a histopathologic analysis. The first study resected the renal tumor immediately after ablation, demonstrating preliminary IRE ablation effect on a cellular level. In this study no definite cell death was observed, implying that IRE effects are not directly established. Wendler et al resected the ablated tumors 4 weeks after the IRE procedure, showing that the ablation zone covered the renal tumors completely. Nonetheless, within the ablation zone very small residues of tumor have been found of uncertain malignancy [22] in 3 cases described. Studies in animal models have demonstrated that the effect of IRE is partially achieved after 3 to 4 weeks [31-33]. Yet resecting ablated RCCs in humans after longer than 4 weeks is not acceptable when ablation is used in curative setting. Hence, the only way to provide insight into the clinical value of these minimal tumor residues is to thoroughly follow patients with intensive imaging studies after IRE ablation. Despite the fact that biopsy during the follow-up targeting the ablation zone may contribute to histopathological confirmation, it would have brought additional burden in a fragile population and would not have been an irrefutable proof of complete ablation. Therefore, as provided by the consensus that ablation success in kidney tumors is assessed by radiological characteristics [30], success in our study will be assessed exclusively based on radiological features.

### Conclusions

In our study, IRE parameters (1500 V/cm, active tip length 15 mm, interelectrode spacing 1 to 2 cm, 90 treatment pulses after 10 sufficient test pulses) were chosen because several studies confirmed that on a histopathological level the ablation zone is completely ablated within the electrode configuration without leaving skip lesions [25,28]. Due to the small sample size and the design of the study, we do not intend to explore different IRE configurations and probe settings.

IRE promises consistent ablation results due to its nonthermal character and is therefore theoretically suitable for centrally located tumors. However, recent literature has investigated the temperature rise of IRE ablation in porcine kidneys and livers, demonstrating a significant temperature rise when repetitive high-intensity pulses are applied [21,34]. Al-Sakere et al showed that when a high amount of energy is applied in a small number of pulses, significant rise in temperature occurs (a phenomenon called Joule heating). In current literature, a solution has been suggested in which the same amount of energy is applied in more pulses, which could result in a mild temperature increase [18,35]. Other factors that can influence the temperature in IRE ablation are varying voltage, pulse length, distance between electrodes, active tip length, and electrode configuration [35]. Furthermore, early clinical practice of IRE in renal tumors close to vital structures demonstrated that no major complications occurred, suggesting that thermal damage of IRE is not clinically significant, and centrally located tumors are suitable for IRE [24].

For the follow-up of renal masses, the most frequently used imaging modality is contrast-enhanced CT. Multiple studies have demonstrated that MRI and CEUS are adequate imaging techniques for follow-up after IRE [36-40]. However, the use of contrast-enhanced CT scan in the follow-up after kidney ablation might be precluded because of potential nephrotoxicity or ionizing radiation exposure in young patients. In the population of patients that receive ablation for their renal mass comorbidity, older age and decrease in renal function are common since their presence entails a clear indication for ablative therapy. Furthermore MRI, applicable to a broader range of the ablated population, may not be easy available and may increase costs. Hence, in this population it is vital to investigate whether other imaging modalities will detect recurrences and residual disease in renal masses with the same accuracy as CT and MRI.

Nononcological outcomes of IRE have been minimally investigated in renal tumors. A total of 2 small studies described serum creatinine levels and demonstrated no significant changes in renal function or transient increase of creatinine which resolved after 1 month [23,24]. Postprocedural pain and length of stay is described by Thomson et al in liver, kidney, and lung (N=36, kidney tumors n=4). While 4 patients were admitted longer than 24 hours, none of these patients had kidney tumors. Postprocedural pain was registered through analgesics use, demonstrating 2 patients who required intravenous or intramuscular analgesics. No patient required prolonged analgesic use after discharge. Quality of life has not been reported in current IRE literature. Insight in nononcological outcomes, including quality of life, is urgently required since treatment decision making is often influenced by this. Particularly in the ablation population, meaning elderly patients with multiple comorbidities, quality of life after an intervention is of great importance.

Categorized as a level 2b study according to the IDEAL classification, this study will provide prospective information on kidney IRE ablation with an extensive description on the radiological evolution of the ablated lesion along time as well as mid-term oncological outcomes. Lastly, we will provide prospective data on quality of life, kidney function, pain level, and duration of admittance after IRE.

## Abbreviations

CEUS: contrast-enhanced ultrasound
CT: computed tomography
CTCAE: Common Terminology Criteria for Adverse Events
DSMB: data safety monitoring board
ECG: electrocardiogram
eGFR: estimated glomerular filtration rate
IDEAL: idea, development, exploration, assessment, long-term study
IRE: irreversible electroporation
IRB: institutional review board
MRI: magnetic resonance Imaging
RFA: radiofrequency ablation
RCC: renal cell carcinoma
SF-36: Short Form 36
SRM: small renal mass
TNM: tumor/node/metastasis
VAS: visual analog scale

## Appendix

Multimedia Appendix 1 Peer-review report IRB (Dutch).

Multimedia Appendix 2 Peer-review report IRB (English).

## References

[1] Chow WH, Devesa SS, Warren JL, Fraumeni JF (1999). Rising incidence of renal cell cancer in the United States. JAMA.

[2] Hollingsworth JM, Miller DC, Daignault S, Hollenbeck BK (2006). Rising incidence of small renal masses: a need to reassess treatment effect. J Natl Cancer Inst.

[3] Jemal A, Siegel R, Xu J, Ward E (2010). Cancer statistics, 2010. CA Cancer J Clin.

[4] Mathew A, Devesa SS, Fraumeni JF, Chow W (2002). Global increases in kidney cancer incidence, 1973-1992. Eur J Cancer Prev.

[5] Volpe A, Cadeddu JA, Cestari A, Gill IS, Jewett MA, Joniau S, Kirkali Z, Marberger M, Patard JJ, Staehler M, Uzzo RG (2011). Contemporary management of small renal masses. Eur Urol.

[6] Wagstaff P, Ingels A, Zondervan P, de la Rosette JJM, Laguna M (2014). Thermal ablation in renal cell carcinoma management: a comprehensive review. Curr Opin Urol.

[7] Katsanos K, Mailli L, Krokidis M, McGrath A, Sabharwal T, Adam A (2014). Systematic review and meta-analysis of thermal ablation versus surgical nephrectomy for small renal tumours. Cardiovasc Intervent Radiol.

[8] Hafron J, Kaouk JH (2007). Ablative techniques for the management of kidney cancer. Nat Clin Pract Urol.

[9] Olweny EO, Cadeddu JA (2012). Novel methods for renal tissue ablation. Curr Opin Urol.

[10] Ljungberg B, Bensalah K, Canfield S, Dabestani S, Hofmann F, Hora M, Kuczyk MA, Lam T, Marconi L, Merseburger AS, Mulders P, Powles T, Staehler M, Volpe A, Bex A (2015). EAU guidelines on renal cell carcinoma: 2014 update. Eur Urol.

[11] Van PH, Becker F, Cadeddu JA, Gill IS, Janetschek G, Jewett MA, Laguna MP, Marberger M, Montorsi F, Polascik TJ, Ukimura O, Zhu G (2011). Treatment of localised renal cell carcinoma. Eur Urol.

[12] Rubinsky B, Onik G, Mikus P (2007). Irreversible electroporation: a new ablation modality—clinical implications. Technol Cancer Res Treat.

[13] Davalos RV, Mir IL, Rubinsky B (2005). Tissue ablation with irreversible electroporation. Ann Biomed Eng.

[14] Lane BR, Moinzadeh A, Kaouk JH (2005). Acute obstructive renal failure after laparoscopic cryoablation of multiple renal tumors in a solitary kidney. Urology.

[15] Johnson DB, Solomon SB, Su L, Matsumoto ED, Kavoussi LR, Nakada SY, Moon TD, Shingleton WB, Cadeddu JA (2004). Defining the complications of cryoablation and radio frequency ablation of small renal tumors: a multi-institutional review. J Urol.

[16] Ogan K, Jacomides L, Dolmatch BL, Rivera FJ, Dellaria MF, Josephs SC, Cadeddu JA (2002). Percutaneous radiofrequency ablation of renal tumors: technique, limitations, and morbidity. Urology.

[17] Deipolyi AR, Golberg A, Yarmush ML, Arellano RS, Oklu R (2014). Irreversible electroporation: evolution of a laboratory technique in interventional oncology. Diagn Interv Radiol.

[18] Al-Sakere B, André F, Bernat C, Connault E, Opolon P, Davalos RV, Rubinsky B, Mir LM (2007). Tumor ablation with irreversible electroporation. PLoS One.

[19] Olweny EO, Kapur P, Tan YK, Park SK, Adibi M, Cadeddu JA (2013). Irreversible electroporation: evaluation of nonthermal and thermal ablative capabilities in the porcine kidney. Urology.

[20] Pech M, Janitzky A, Wendler JJ, Strang C, Blaschke S, Dudeck O, Ricke J, Liehr U (2011). Irreversible electroporation of renal cell carcinoma: a first-in-man phase I clinical study. Cardiovasc Intervent Radiol.

[21] Wagstaff PGK, de Bruin DM, van den Bos W, Ingels A, van Gemert MJC, Zondervan PJ, Verdaasdonk RM, van Lienden KP, van Leeuwen TG, de la Rosette JJM, Laguna Pes MP (2015). Irreversible electroporation of the porcine kidney: temperature development and distribution. Urol Oncol.

[22] Wendler JJ, Porsch M, Nitschke S, Köllermann J, Siedentopf S, Pech M, Fischbach F, Ricke J, Schostak M, Liehr UB (2015). A prospective Phase 2a pilot study investigating focal percutaneous irreversible electroporation (IRE) ablation by NanoKnife in patients with localised renal cell carcinoma (RCC) with delayed interval tumour resection (IRENE trial). Contemp Clin Trials.

[23] Trimmer CK, Khosla A, Morgan M, Stephenson SL, Ozayar A, Cadeddu JA (2015). Minimally invasive percutaneous treatment of small renal tumors with irreversible electroporation: a single-center experience. J Vasc Interv Radiol.

[24] Thomson KR, Cheung W, Ellis SJ, Federman D, Kavnoudias H, Loader-Oliver D, Roberts S, Evans P, Ball C, Haydon A (2011). Investigation of the safety of irreversible electroporation in humans. J Vasc Interv Radiol.

[25] Wendler JJ, Ricke J, Pech M, Fischbach F, Jürgens J, Siedentopf S, Roessner A, Porsch M, Baumunk D, Schostak M, Köllermann J, Liehr U (2016). First delayed resection findings after irreversible electroporation (IRE) of human localised renal cell carcinoma (RCC) in the IRENE pilot phase 2a trial. Cardiovasc Intervent Radiol.

[26] McCulloch P, Altman DG, Campbell WB, Flum DR, Glasziou P, Marshall JC, Nicholl J, Balliol C, Aronson JK, Barkun JS, Blazeby JM, Boutron IC, Campbell WB, Clavien P, Cook JA, Ergina PL, Feldman LS, Flum DR, Maddern GJ, Nicholl J, Reeves BC, Seiler CM, Strasberg SM, Meakins JL, Ashby D, Black N, Bunker J, Burton M, Campbell M, Chalkidou K, Chalmers I, de LM, Deeks J, Ergina PL, Grant A, Gray M, Greenhalgh R, Jenicek M, Kehoe S, Lilford R, Littlejohns P, Loke Y, Madhock R, McPherson K, Meakins J, Rothwell P, Summerskill B, Taggart D, Tekkis P, Thompson M, Treasure T, Trohler U, Vandenbroucke J (2009). No surgical innovation without evaluation: the IDEAL recommendations. Lancet.

[27] Nielsen K, Scheffer HJ, Vieveen JM, van Tilborg AAJM, Meijer S, van KC, van den Tol MP, Meijerink MR, Bouwman RA (2014). Anaesthetic management during open and percutaneous irreversible electroporation. Br J Anaesth.

[28] van den Bos W, de Bruin DM, Jurhill RR, Savci-Heijink CD, Muller BG, Varkarakis IM, Skolarikos A, Zondervan PJ, Laguna-Pes MP, Wijkstra H, de Reijke TM, de la Rosette JJM (2016). The correlation between the electrode configuration and histopathology of irreversible electroporation ablations in prostate cancer patients. World J Urol.

[29] Schneider M (1999). Characteristics of SonoVue. Echocardiography.

[30] Zondervan PJ, Wagstaff PGK, Desai MM, de Bruin DM, Fraga AF, Hadaschik BA, Köllermann J, Liehr UB, Pahernik SA, Schlemmer HP, Wendler JJ, Algaba F, de la Rosette JJM, Laguna Pes MP (2016). Follow-up after focal therapy in renal masses: an international multidisciplinary Delphi consensus project. World J Urol.

[31] Deodhar A, Monette S, Single GW, Hamilton WC, Thornton R, Maybody M, Coleman JA, Solomon SB (2011). Renal tissue ablation with irreversible electroporation: preliminary results in a porcine model. Urology.

[32] Wendler JJ, Porsch M, Hühne S, Baumunk D, Buhtz P, Fischbach F, Pech M, Mahnkopf D, Kropf S, Roessner A, Ricke J, Schostak M, Liehr U (2013). Short- and mid-term effects of irreversible electroporation on normal renal tissue: an animal model. Cardiovasc Intervent Radiol.

[33] Tracy CR, Kabbani W, Cadeddu JA (2011). Irreversible electroporation (IRE): a novel method for renal tissue ablation. BJU Int.

[34] Faroja M, Ahmed M, Appelbaum L, Ben-David E, Moussa M, Sosna J, Nissenbaum I, Goldberg SN (2013). Irreversible electroporation ablation: is all the damage nonthermal?. Radiology.

[35] van den Bos W, Scheffer HJ, Vogel JA, Wagstaff PGK, de Bruin DM, de Jong MC, van Gemert MJC, de la Rosette JJM, Meijerink MR, Klaessens JH, Verdaasdonk RM (2016). Thermal energy during irreversible electroporation and the influence of different ablation parameters. J Vasc Interv Radiol.

[36] Mahmood F, Hansen RH, Agerholm-Larsen B, Jensen KS, Iversen HK, Gehl J (2011). Diffusion-weighted MRI for verification of electroporation-based treatments. J Membr Biol.

[37] Padia SA, Johnson GE, Yeung RS, Park JO, Hippe DS, Kogut MJ (2016). Irreversible electroporation in patients with hepatocellular carcinoma: immediate versus delayed findings at MR Imaging. Radiology.

[38] van den Bos W, de Bruin DM, van Randen A, Engelbrecht MRW, Postema AW, Muller BG, Varkarakis IM, Skolarikos A, Savci-Heijink CD, Jurhill RR, Zondervan PJ, Laguna Pes MP, Wijkstra H, de Reijke TM, de la Rosette JJM (2016). MRI and contrast-enhanced ultrasound imaging for evaluation of focal irreversible electroporation treatment: results from a phase I-II study in patients undergoing IRE followed by radical prostatectomy. Eur Radiol.

[39] Wiggermann P, Zeman F, Niessen C, Agha A, Trabold B, Stroszczynski C, Jung EM (2012). Percutaneous irreversible electroporation (IRE) of hepatic malignant tumours: contrast-enhanced ultrasound (CEUS) findings. Clin Hemorheol Microcirc.

[40] Niessen C, Jung EM, Beyer L, Pregler B, Dollinger M, Haimerl M, Scheer F, Stroszczynski C, Wiggermann P (2015). Percutaneous irreversible electroporation (IRE) of prostate cancer: Contrast-enhanced ultrasound (CEUS) findings. Clin Hemorheol Microcirc.

