# Supplementary material for: Irreversible Electroporation for the Ablation of Renal Cell Carcinoma: A Prospective, Human, In Vivo Study Protocol (IDEAL Phase 2b)
Source: JMIR Res Protoc. 2017 Feb 16;6(2):e21. doi: 10.2196/resprot.6725 (PMC5334515; doi:10.2196/resprot.6725)
Supplement: Multimedia Appendix 1 [file resprot_v6i2e21_app1.pdf]

Academisch Medisch Centrum
Universiteit van Amsterdam

Aan mevrouw prof.dr. M.P. Laguna Pes
Urologie
G4-163

Amsterdam, 9 mei 2016
ons kenmerk: 2016_055#B2016313
betreft: **Positief besluit**
NL56935.018.16
The safety and feasibility of irreversible electroporation for the ablation of small renal masses: a prospective in-vivo pilot study

Medisch Ethische Toetsingscommissie
E2-172
telefoon: 020 56 67389

Geachte mevrouw Laguna Pes,

De METC AMC heeft zich, op grond van artikel 2, lid 2, sub a van de Wet medisch-wetenschappelijk onderzoek met mensen (WMO) beraden over bovengenoemd onderzoeksdossier.
Wij delen u gaarne mee dat onze commissie
- tot oordelen bevoegd krachtens artikel 2, tweede lid, onder a, van de Wet medisch-wetenschappelijk onderzoek met mensen (WMO);
- werkzaam volgens de ICH-GCP richtlijnen;
- op grond van de haar voorgelegde stukken als hierna vermeld;
- gelet op artikel 3 van de WMO;
- gelet op artikel 5 en 6;
- vastgesteld hebbende dat voorzien is in de dekking van een aansprakelijkheidsverzekering als bedoeld in artikel 7, lid 9 van de WMO,

heeft besloten tot een positief oordeel over deze studie en de uitvoering daarvan in het AMC.

Voorts hebben wij vastgesteld dat voor het onderzoek een verzekering is afgesloten conform de WMO door AMC ten behoeve van proefpersonen van dit onderzoek.

In de beoordeling betrokken documenten:
A1 aanbiedingsbrief d.d. 22 februari 2016
A1 aanbiedingsemail d.d. 22 februari 2016
A1 aanbiedingsemail d.d. 22 april 2016
A1 aanbiedingsemail d.d. 4 mei 2016
B1 ABR-formulier NL56935.018.16 versie 02 d.d. 22 april 2016
B2 AMC appendix, getekend d.d. 22 april 2016
C1 protocol versie 3 d.d. 4 mei 2016 TC
D1 IB NanoKnife V1 IRE for SRMs versie 2.0 d.d. 8 april 2011
D4 certificaat NanoKnife CE 559984 V1 IRE for SRMs d.d. 27 maart 2012
E1 E2 proefpersoneninformatie en toestemmingsverklaring versie 3 d.d. 4 mei 2016 TC
H1 CV onafh. arts Klümpen, AMC
I3 CV hoofdonderzoeker Laguna Pes, AMC
K5 DSMB charter versie 2 d.d. 22 april 2016

Het onderzoeksdossier, aan ons ter beoordeling voorgelegd op 22 februari 2016 is besproken in de vergadering van onze commissie van 3 maart 2016. Er zijn vragen gesteld over onder andere de risico's van de behandeling en de effectiviteit. Vervolgens is het aan de orde geweest in de vergadering van het dagelijks bestuur van onze commissie van 3 mei 2016 na ontvangst van aangepaste stukken op 22 april 2016. Daarbij bleken de vragen voldoende beantwoord en werd de afhandeling gemandateerd aan de secretaris. Deze heeft vastgesteld dat na de aanbieding van gewijzigde documenten op 4 mei 2016 voldoende tegemoet gekomen is aan de opmerkingen van de commissie.

Meibergdreef 9 Postbus 22660 1100 DD Amsterdam Telefoon (020) 566 9111 Fax (020) 566 4440

U dient onze commissie op de hoogte te stellen van de daadwerkelijke start van het onderzoek, van de (al dan niet voortijdige) beëindiging daarvan, en van tijdens de studie optredende onverwachte complicaties. Voorts dienen eventuele protocolwijzigingen ter beoordeling aan onze commissie te worden voorgelegd. U dient tevens ons jaarlijks een voortgangsrapportage betreffende de studie te doen toekomen, voor het eerst binnen een jaar na dagtekening van dit besluit.

Wij wijzen u erop dat op grond van artikel 23 van de Wet medisch-wetenschappelijk onderzoek met mensen degene wiens belang rechtstreeks bij een besluit van de METC is betrokken, daartegen binnen zes weken na de dag waarop het besluit bekend is gemaakt, een administratief beroepschrift kan indienen bij de Centrale Commissie Mensgebonden Onderzoek. Een dergelijk administratief beroepschrift dient geadresseerd te worden aan: CCMO, Postbus 16302, 2500 BH Den Haag.

Tenslotte brengen wij onder uw aandacht dat dit besluit zijn geldigheid verliest als de studie niet binnen één jaar na dagtekening van deze brief is gestart.

Ten tijde van de beoordeling van dit project was de commissie als volgt samengesteld:

prof.dr. M.P.M. Burger	:	voorzitter, gynaecoloog
mw.drs. G.H.M. van Ammers	:	lid dat onderzoek beoordeelt vanuit de invalshoek van de proefpersoon
drs. P.M. Bet	:	plv. lid ziekenhuisapotheker, klinisch farmacoloog
mw.prof.dr. M.A. Boermeester	:	chirurg
dr. A.J. Bredenoord	:	maag darm lever arts
dr. M.G.W. Dijkgraaf	:	plv. lid, methodoloog
mw. J.M.M. Dijkstra	:	lid dat onderzoek beoordeelt vanuit de invalshoek van de proefpersoon
dr. M. Figee	:	psychiater
prof.dr. R.C.M. Hennekam	;	hoogleraar kindergeneeskunde en translationele genetica
prof.dr. J.J. Homan van der Heide	:	internist
dr. J.M.N.E. Jans	:	plv. lid, ethicus
dr. J.Ph. de Jong	:	plv. lid, ethicus
dr. R.E. Jonkers	:	longarts/plv. lid klinisch farmacoloog
prof.dr. A.H.L.C. van Kaam	:	kinderarts
mw.dr. E.M. Kemper	:	plv. lid ziekenhuisapotheker, klinisch farmacoloog
dr. M.J.W. Koelemay	:	vaatchirurg
mw.dr. S.J. de Kort	:	plv. lid, medisch ethicus
prof.mr.dr. J. Legemaate	:	plv. lid, jurist
prof.dr. R.A.A. Mathôt	:	ziekenhuisapotheker, klinisch farmacoloog
dr. G.A. van Montfrans	:	internist
dr. P.J Nederkoom	:	neuroloog
prof.dr. M. Nieuwdorp	:	internist
mw.mr.dr. M.C. Ploem	:	gezondheidsjurist
dr. G. ter Riet	:	plv. lid methodoloog
prof.dr. A.J.P.M. Smout	:	maag darm lever arts
mw.mr. L.M. Spittuler	:	lid dat onderzoek beoordeelt vanuit de invalshoek van de proefpersoon
dr. H.L. Tan	:	cardioloog
prof.dr. J.G.P. Tijssen	:	klinisch epidemioloog
mw.prof.dr. S. van de Vathorst	:	plv. lid, medisch ethicus
prof.dr. M. Vermeulen	:	neuroloog
mw.dr. A.M. Westermann	:	internist-oncoloog
prof.dr. D.L. Willems	:	medisch ethicus
prof.dr. A.H. Zwinderman	:	biostatisticus.

Met vriendelijke groet,
namens de Medisch Ethische Toetsingscommissie,

Mw. dr. C.L. van der Wilt
ambtelijk secretaris

c.c. CCMO (pdf via TOL)
c.c. AMC Medical Research BV (pdf per e-mail) (+)
c.c. pdf per e-mail M.Buijs
c.c. EPIC (registratie)
