# Supplementary material for: Irreversible Electroporation for the Ablation of Renal Cell Carcinoma: A Prospective, Human, In Vivo Study Protocol (IDEAL Phase 2b)
Source: JMIR Res Protoc. 2017 Feb 16;6(2):e21. doi: 10.2196/resprot.6725 (PMC5334515; doi:10.2196/resprot.6725)
Supplement: Multimedia Appendix 2 [file resprot_v6i2e21_app2.pdf]

Academisch Medisch Centrum

Universiteit van Amsterdam

Professor M.P. Laguna Pes
Department of Urology
G4-163

Medical Ethics Review Committee
E2-170
tel: 0031 20 566 1358

Amsterdam, July 12, 2016
Your reference:
Our reference: METC 2016_055#B2016313ENG
Subject:
METC 2016_055 Positive judgement concerning your project:
NL56935.018.16
The safety and feasibility of irreversible electroporation for the ablation of small renal masses: a prospective in-vivo pilot study

Dear Professor Laguna Pes,

On request of Ms. M. Buijs, I hereby confirm on behalf of the Medical Ethics Review Committee of the AMC that our committee approved the above mentioned research project on May 9, 2016.

For a list of documents on which the approval has been based and for a list of members of our committee at the time of the approval I refer to our original approval letter METC 2016_055#B2016313.

The following center was approved to participate:
- AMC (date of approval: May 9, 2016)

Our committee has jurisdiction to decide on behalf of article 2, second paragraph, under a, of the Medical Research involving Human Subjects Act (WMO) and operates in agreement with ICH-GCP guidelines.

Yours sincerely,
on behalf of the Medical Ethics Review Committee of the Academic Medical Center,

Mrs. T. Groenveld
secretary

Appendix: letter approval METC 2016_055#B2016313.

c.c. per email: M.Buijs
